# Bioinformatic data processing pipelines in support of next‐generation sequencing‐based HIV drug resistance testing: the Winnipeg Consensus

**DOI:** 10.1002/jia2.25193

**Published:** 2018-10-22

**Authors:** Hezhao Ji, Eric Enns, Chanson J. Brumme, Neil Parkin, Mark Howison, Emma R. Lee, Rupert Capina, Eric Marinier, Santiago Avila‐Rios, Paul Sandstrom, Gary Van Domselaar, Richard Harrigan, Roger Paredes, Rami Kantor, Marc Noguera‐Julian

**Affiliations:** ^1^ National HIV and Retrovirology Laboratories at JC Wilt Infectious Diseases Research Centre Public Health Agency of Canada Winnipeg MB Canada; ^2^ Department of Medical Microbiology and Infectious Diseases University of Manitoba Winnipeg MB Canada; ^3^ Bioinformatics Core at the National Microbiology Laboratory Public Health Agency of Canada Winnipeg MB Canada; ^4^ British Columbia Centre for Excellence HIV/AIDS Vancouver BC Canada; ^5^ Data First Consulting Inc. Belmont CA USA; ^6^ Watson Institute for International and Public Affairs Brown University Providence RI USA; ^7^ Centre for Research in Infectious Diseases National Institute of Respiratory Diseases Mexico City Mexico; ^8^ Division of AIDS Department of Medicine University of British Columbia Vancouver BC Canada; ^9^ IrsiCaixa AIDS Research Institute Badalona Catalonia Spain; ^10^ Division of Infectious Diseases Brown University Alpert Medical School Providence RI USA

**Keywords:** next‐generation sequencing, HIV drug resistance test, bioinformatics, pipeline, Winnipeg Consensus, guideline

## Abstract

**Introduction:**

Next‐generation sequencing (NGS) has several advantages over conventional Sanger sequencing for HIV drug resistance (HIVDR) genotyping, including detection and quantitation of low‐abundance variants bearing drug resistance mutations (DRMs). However, the high HIV genomic diversity, unprecedented large volume of data, complexity of analysis and potential for error pose significant challenges for data processing. Several NGS analysis pipelines have been developed and used in HIVDR research; however, the absence of uniformity in data processing strategies results in lack of consistency and comparability of outputs from different pipelines. To fill this gap, an international symposium on bioinformatic strategies for NGS‐based HIVDR testing was held in February 2018 in Winnipeg, Canada, convening laboratory scientists, bioinformaticians and clinicians involved in four recently developed, publicly available NGS HIVDR pipelines. The goal of this symposium was to establish a consensus on effective bioinformatic strategies for NGS data management and its use for HIVDR reporting.

**Discussion:**

Essential functionalities of an NGS HIVDR pipeline were divided into five analytic blocks: (1) NGS read quality control (QC)/quality assurance (QA); (2) NGS read alignment and reference mapping; (3) HIV variant calling and variant QC; (4) NGS HIVDR reporting; and (5) extended data applications and additional considerations for data management. The consensuses reached among the participants on all major aspects of these blocks are summarized here. They encompass not only recommended data management and analysis strategies, but also detailed bioinformatic approaches that help ensure accuracy of the derived HIVDR analysis outputs for both research and potential clinical use.

**Conclusions:**

While NGS is being adopted more broadly in HIVDR testing laboratories, data processing is often a bottleneck hindering its generalized application. The proposed standardization of NGS read QC/QA, read alignment and reference mapping, variant calling and QC, HIVDR reporting and relevant data management strategies in this “Winnipeg Consensus” may serve as a starting guideline for NGS HIVDR data processing that informs the refinement of existing pipelines and those yet to be developed. Moreover, the bioinformatic strategies presented here may apply more broadly to NGS data analysis of microbes harbouring significant genomic diversity.

## 1 Introduction

Successful antiretroviral therapy (ART) suppresses HIV viral load, reduces the incidence of new infections and increases the life expectancy of infected individuals 1, 2, 3, 4, 5. However, HIV drug resistance (HIVDR) can occur as result solely from poor proof‐reading during viral replication or the combined effect from poor proof‐reading and drug selection during unsuccessful ART 6, 7. With drastic increase in ART coverage worldwide, HIVDR has become a major barrier that hinders its effectiveness 8. Conventional HIVDR genotyping qualitatively detects drug resistance mutation (DRM) using Sanger sequencing approaches, which has limited capacity in reliable detection of minority variants present at frequencies below approximately 20%, with potentially relevant clinical impact 9, 10, 11.

Next‐generation sequencing (NGS), as exemplified by Illumina sequencing‐by‐synthesis technology, refers to newer sequencing technologies that enable high‐throughput, massively parallel sequencing of individual input templates 11, 12, 13. When applied to HIVDR genotyping, such technologies bestow unique advantages and significantly improve sensitivity for resolving complex HIV quasispecies with exceptional resolution and quantitative minority variant identification 11, 13, 14. The high scalability and ongoing cost reduction of NGS also permit further improvement in time efficiency and cost‐effectiveness of NGS HIVDR assays when many batched specimens are being processed 15, 16, 17. While broader adoption in testing laboratories could lead to new NGS‐based standards for HIVDR genotyping, some important issues remain to be addressed, including lack of standardization for NGS HIVDR data analysis pipelines and resulting accurate and meaningful low‐abundance variant data interpretation 11, 13, 18.

Like other molecular assays, the routine use of NGS HIVDR assays requires fully validated protocols that dictate sample processing in the laboratory. However, NGS also requires well‐defined bioinformatics strategies and tools that help to reliably convert raw NGS data into user‐interpretable HIVDR results. Notably, with the broad adoption of NGS, the sequencing itself has become relatively less challenging, while the data processing steps have become the primary bottleneck for its generalized application to HIVDR. Such challenges arise largely from: (1) high HIV sequence diversity 19; (2) unprecedented large volume of NGS data, (3) sequence‐specific errors, some of which are intrinsic to different NGS platforms 20, 21; (4) relatively short NGS read lengths with suboptimal basecalling accuracies; and (5) requirement for advanced bioinformatics skills and high performance computing capacity. Most NGS software applications are designed for the analysis of organismal genomes of a fixed ploidy and having modest sequence coverage. In contrast, the HIV genome exists as a quasispecies, and thus presents unique challenges for its sequencing and analysis. Existing NGS analysis pipelines for HIVDR to date have been developed by independent research groups with little coordination or any pre‐existing guidelines to reference, and thus differ in their data processing strategies and their output formats (Table 1). This lack of conventions to which to adhere leads to uncertainties in data reliability and also makes the comparison of outputs from different pipelines unnecessarily difficult 11. Moreover, it also impedes the ability of regulatory agencies to standardize and benchmark such assays for accreditation purposes. Thus, a consensus recommendation on standards for bioinformatic analysis and reporting conventions for HIVDR research and clinical purposes is urgently required.

**Table 1:** Currently available pipeline/software for automated NGS‐based HIVDR data analysis

Pipeline/software	Reference information		Resources			Technical characteristics			HIV drug resistance		HIVDR data analysis features		
	URL	Year^c^	Cost^d^	Time^e^	Bioinformatic IT needs^f^	Compatible NGS platform	Cloud based	Web interface	Designed for HIVDR	Ref DB and algorithm^g^	Output (nt/*aa*)^h^	QA checks[Link]	InDel^j^
V‐Phaser 2 40	https://www.broadinstitute.org/viral‐genomics/v‐phaser‐2	2013	Free	N/A	Yes	N/A	No	No	No	No	csv/*csv*	E	Yes
ShoRah 41	https://github.com/cbg‐ethz/shorah	2013	Free	N/A	Yes	N/A	No	No	No	No	csv/*N/A*	E	N/A
VirVarSeq 42	https://sourceforge.net/projects/virtools/	2015	Free	N/A	Yes	Illumina	No	No	No	No	fasta/*csv*	Q/E	Yes
MinVar 43	https://ozagordi.github.io/MinVar/	2016	Free	<1 hour	Yes	Illumina	Yes^k^	No	Yes	HIVdb	vcf/*csv*	Q	Yes
V‐pipe	https://cbg‐ethz.github.io/V‐pipe/	2017	Free	N/A	Yes	Illumina	No	No	No	No	fasta/csv	Q	Yes
Hivmmer 44	https://github.com/kantorlab/hivmmer	2017	Free	<1 hour	Yes	Illumina	No	No	Yes	No	csv/*csv*	L	Yes
Geno2Pheno[ngs‐freq]^45^ ^a^	http://ngs.geno2pheno.org/	2018	Free	<1 minute	Yes	N/A	No	Yes	Yes	g2p[res]	fasta/*csv*	N/A	Yes
MiCall^b^	https://github.com/cfe‐labMiCall	2016	Free	<1 hour	No	Illumina	Yes	Yes	Yes	HIVdb	Csv/*csv*	Q/E	Yes
HyDRA	https://hydra.canada.ca	2016	Free	<1 hour	No	Illumina Ion Torrent	No	Yes	Yes	HIVdb	fasta,vcf/*aavf*	Q/E	Yes
PASeq.org	https://www.paseq.org	2016	Free	<1 hour	No	Illumina	Yes	Yes	Yes	HIVdb	fasta/*csv*	Q/C/A	Yes
DeepChek HIV 46	https://www.ablsa.com/overview/deepchek/	2014	$65^l^	<1 hour	No	Illumina Ion Torrent	Yes	Yes	Yes	HIVdb	csv/*csv*	Q	Yes
Smart GeneHIV	http://www.smartgene.com/mod_ngs.html	2016	N/A	N/A	No	N/A	No	Yes	Yes	HIVdb	N/A	N/A	Yes
Vela sentosa HIV 47	http://www.veladx.com/HIV.html	2016	$200^m^	N/A	No	Ion Torrent	No	Yes	Yes	HIVdb	fasta/*csv*	Q	Yes
Hyrax Exatype	https://exatype.com/	2018	N/A	<1 hour	No	Illumina IonTorrent	Yes	Yes	Yes	HIVdb	fasta/*csv*	Q	Yes

The pipelines/software are categorized as: (1) freely available software for bioinformaticians (top block); (2) freely available software suitable for non‐bioinformaticians (middle block); and (3) commercial software (bottom block). Within each block, the chronological order was followed. N/A**,** Information not available or not applicable. NGS, next‐generation sequencing; HIVDR, HIV drug resistance.

^a^Geno2pheno[ngs‐freq] pipeline can only use a codon frequency table as an input which needs to be obtained separately; ^b^pending approval and release on Illumina BaseSpace Sequence Hub. For early access, please micalldev@cfenet.ubc.ca; ^c^year of publication/*public availability*; ^d^approximate per sample cost of bioinformatic data analysis only; ^e^time range for single sample data analysis (data transfer time excluded); ^f^refers to the need of on‐site computational infrastructure or expert staff; ^g^Ref DB and algorithm: reference HIV resistance database and/or algorithms for HIV resistance interpretation *(HIVdb: Stanford HIV Database)*; g2p[res] refers to the Geno2pheno[resistance] statistical engine; ^h^output: format of output files reporting nucleotide (nt) and amino acid (aa) variations; ^i^QA check strategies incorporated for NGS read quality assurance (Q: Quality Control; C: Contamination Control; E: Sequencing Error Model; L: Alignment Quality Filter; A: ApoBEC Hypermutation Detection); ^j^indels are recognizable by default but no codon‐aware strategies are implemented for reporting insertion/deletion mutations specifically associated to HIV resistance; ^k^can be ported to Cloud; ^l^cost based on general access through Illumina basespace; ^m^approximate cost of whole sample analysis (sample preparation, sequencing, data analysis).

Development of such a consensus necessitates knowledge of NGS data characteristics, relevant bioinformatics skill sets, appreciation of the clinical relevance (or lack thereof) of minority variants and, importantly, extensive expertise and experience in performing NGS HIVDR data analysis. In this commentary, we report the outcome of an international symposium on bioinformatic strategies for NGS HIVDR testing, which was held in February 2018 in Winnipeg, Canada, convening bioinformaticians, scientists and clinicians from four NGS HIVDR pipeline teams, including: HyDRA from the National Microbiology Laboratory in Canada, PASeq.org from Institute for AIDS Research (IrsiCaixa) in Spain, MiCall from the British Columbia Centre for Excellence in HIV/AIDS in Canada and hivmmer from the Providence‐Boston Center for AIDS Research at Brown University in USA. Notably, HyDRA, PASeq.org and MiCall are freely available web interfaces and are used by many investigators worldwide, while hivmmer and several other pipelines are also freely available but still require advanced computational skills to execute (Table 1). In‐depth discussions and brainstorming sessions were organized during the symposium. The consensus for NGS‐based HIVDR data analysis that was reached among the participating groups (referred to as the “Winnipeg Consensus” hereafter) is summarized and presented here. It is noteworthy that all bioinformatics strategies discussed at the symposium and presented in this “Winnipeg Consensus” are based on the second‐generation sequencing technologies exemplified by Illumina sequence‐by‐synthesis technology.

## 2 Discussion

The characteristics of an optimal NGS HIVDR data processing pipeline include: (1) automated data analysis with a short turnaround time; (2) accommodation of all relevant HIV genes and raw data from varied NGS platforms; (3) incorporation of essential quality assurance (QA)/quality control (QC) strategies to ensure data accuracy and reproducibility; (4) production of customizable and easy‐to‐interpret HIVDR reports that satisfy research, surveillance and clinical monitoring needs; (5) user‐friendliness requiring minimal or no bioinformatics experience; and (6) easy access with minimal additional cost to the end‐users. The Winnipeg Consensus covers the major bioinformatic strategies that help to satisfy these requirements.

Although pipelines vary, some basic principles apply in NGS HIVDR data analysis. The analytic components of an NGS HIVDR pipeline were grouped into five sequential functional blocks: (1) NGS read QC/QA; (2) NGS read alignment and reference mapping; (3) HIV variant calling and variant QC; (4) HIVDR interpretation and reporting; and (5) analysis data management. Table 2 details the Winnipeg Consensus on the major functionalities in each of these blocks, including analysis objectives, consensus on strategies and associated considerations, where applicable. The highlights include:

**Table 2:** Outcomes of the Winnipeg Consensus for recommended bioinformatic strategies for an NGS‐based HIVDR data analysis pipeline

Functional blocks	Objectives	Consensus on strategies	Notes and comments
1. NGS read quality control/quality assurance	To ensure only quality reads are applied in downstream data processing	1. Average quality score (QS) of the read: 25	A QS at 25 corresponds to an estimated sequencing error rate of 0.3% 48. When possible, direct QS examination for all individual bases and exclusion of those with scores <25 from subsequent analysis are recommended
		2. Minimum read length: 75 bases	This is based on Illumina 300‐cycle paired‐end sequencing and it may vary if another NGS platform or sequencing protocol is applied
		3. Contamination check: recommended	External non‐viral contamination may be interfering with HIV NGS efficiency. HIV cross‐sample contamination or “index hopping” implies errors in laboratory sample processing which may lead to erroneous minority variant detection (see strategies implemented in V‐Pipe and ViCroSeq[https://github.com/mnoguera/ViCroSeq] tools) 49
		4. APOBEC mutation check: recommended	Presence of APOBEC‐edited DNA templates in the sequenced sample may result in the artefactual detection of minority variant related to APOBEC activity. Filtering this non‐viable sequence content is beneficial especially when significant amounts of HIV proviral DNA may be present in the input specimen (i.e. PBMC, dried blood spots) 50, 51
2. NGS read alignment and reference mapping	To ensure the efficiency and accuracy of NGS read alignment to reference sequences	1. HIV‐1 reference: HXB‐2	For conserved regions such as HIV‐1 *pol*, the choice of reference has minimal impact on subsequent alignment to a single reference. HXB‐2 is a natural choice for the reference sequence since it provides the standard coordinate system for reporting DRMs. Iterative realignment to a sample‐specific consensus may also reduce the importance of the initial choice of reference sequence. However, for variable regions such as *env*, a more comprehensive collection/database of reference sequences should be evaluated
		2. NGS read aligner: short‐read aligner is recommended	Bowtie2 is thus far the most commonly used NGS short‐read aligner due to its speed, availability, documentation, ease of installation and active maintenance 26. An alternative to NGS short‐read alignment is to conduct probabilistic multiple‐sequence alignment with HMMER 52. Other aligners and alignment strategies that have been previously evaluated by the group but are no longer in use include SMALT, BWA‐MEM, BLAST 53, custom implementations of codon‐aware Smith–Waterman alignment 54, MOSAIK 55, stampy 56 and SHRiMP2 57
		3. Analysis of whole *pol* gene: required	Coverage of the entire *pol* region is required to enable HIVDR analysis on all genes encoding the three ART‐targeted enzymes (protease, reverse transcriptase and integrase)
		4. Indel management: required	Effective indel management strategy (i.e. codon‐aware alignments) is not available in existing pipelines. However, with several indel variations contributing to HIVDR, full‐codon indels should be properly identified and reported
3. HIV variant calling and variant quality control	To ensure the accuracy of variant calling	1. QC/QA of nucleotide variant calling: recommended	Additional QA/QC procedures may be incorporated to further ensure the variant call accuracy. For instance, HyDRA calls variation only when minimum allele counts is ≥5, minimum QS of variant allele is ≥30 and read depth at the relevant variation site is ≥100
		2. Amino acid variation calling based on NGS reads, but NOT consensus sequence: required	Consensus sequence‐based DRM analysis renders inevitable assumption while ≥2 mixed bases present in the codon, which diminishes NGS values in quantitative DRM detections
		3. Secondary QC for minority variant calling: optional	It helps to exclude erroneous variant calls via statistical estimation based on gross platform‐specific error rate, as determined by parallel sequencing of pedigreed plasmid in the NGS run, which sums all potential errors from any involved assay procedures
4. HIVDR interpretation and reporting	HIVDR interpretation	Query reference database and algorithms: HIVdb (https://hivdb.stanford.edu)	Although minor discrepancies exist with other alike databases, Stanford database (HIVdb) is recommended for better general adoption
	Concise report *(for potential clinical use)*	The report should contain the following: Patient and sample information if provided (optional) Exportable/printable HIVDR report with DRMs and colour‐coded resistance interpretations Two‐column summary on DRMs at reporting threshold of 5% and 15% respectively with no detailed frequencies Pipeline and software version applied Comment on the accreditation status of the assay for clinical use	Concise HIVDR reports from NGS data should simulate Sanger sequencing output for easier adoption and interpretation by clinicians, to be used for clinical care Integrase gene should be examined in addition to reverse transcriptase and protease and samples with no integrase data should be flagged The reporting thresholds are suggested to simulate sensitivity of SS in DRM detection (15%) and to exemplify a practical threshold for reporting authentic DRMs of potential clinical relevance with minimal interference from errors/bias (5%). Further refinement of these values may be required as relevant research advances
	Comprehensive report *(for research use)*	The report should contain the following: All contents included in clinical reports. Summary on filtering statistics, quality metrics and coverage plots Quantitative report on all HIV‐1 DRMs with exact frequencies Consensus sequence with threshold of 15% for mixed base call	Comprehensive reports should contain all NGS‐derived data that researchers may utilize for various application purposes Customizable HIVDR reporting is encouraged to enable users to construct report that best serves their needs. For instance, a customizable frequency threshold setting for DRM identification and reporting and user‐definable threshold(s) for consensus sequence generation are recommended
	Other exportable data *(Optional)*	Consensus sequences with user‐defined threshold: recommended Variant reports on all nucleotide loci: recommended Variant reports on all amino acid loci: recommended Codon usage at all amino acid variations loci: recommended	Standard VCF/BCF format is recommended for nucleotide variant reports. To facilitate comparisons and merging of data from different pipelines, a new standard .aavf reporting format is proposed (Appendix* * 1 *,* https://github.com/winhiv/aavf-spec). A tool suite to parse aavf format is available at https://github.com/winhiv/PyAAVF. The aavf file provides an amino acid variation summary, along with frequencies of relevant codons, across the examined region based on the associated NGS reads directly. It may serve as a generic variation report template from any NGS analysis
5. General Analysis data management	Data storage	Raw NGS data: to be stored by data generator while data analysis providers may transiently store it for reviewing purpose Intermediate files *(e.g. SAM, BAM)*: no need to store Versioning data files for the applied pipeline: recommended	Automated versioning of all analysis results, reports and intermediate data files is required for retroactive data assessments when necessary
	Data disposal	Analysis provider disposes data after a defined holding period	Deposition of data into public archives (e.g. NCBI Sequence Read Archive) requires informed consent from the data generator
	Data ownership	Policy varies among different institutes and countries Clear data ownership statement should be included in Terms of Service and Conditions	In general, data generators own all the data while unidentified data may be used by data analysis provider for evaluation or research purposes providing mutual agreement is in place

DRM, drug resistance mutation; NGS, next‐generation sequencing; PBMC, peripheral blood mononuclear cell.

1. “**NGS read QC/QA**” warrants that only high‐quality NGS reads are to be utilized in downstream HIVDR data analysis. Although all NGS platforms attach quality scores to individual basecalls, the additional NGS read QC/QA steps described in this consensus were deemed both necessary and effective in reducing false variant calling. Only basic read QC/QA strategies are described here and more stringent filtering may be required in certain cases.
2. “**NGS read alignment and reference mapping**” addresses the needs for valid and accurate read alignment to designated reference sequence(s) that enables subsequent variant calling. Pipelines should at minimum support reference mapping of the whole HIV *pol* gene, which encodes the three main drug‐targeted HIV enzymes: protease (PR), reverse transcriptase (RT) and integrase (IN). Although not urgently required for HIVDR genotyping, it would be beneficial for pipelines to also accommodate *full‐length* HIV reference alignment, since many users are adopting NGS for partial or full‐length HIV sequencing beyond the *pol* gene. Notably, genetic variability in the HIV *env* gene poses more challenges for reference alignment strategies than the relatively conserved *pol* gene. Certain insertions and deletions (indels) in HIV‐1 PR (near codon 35) and RT (near codon 69) genes are associated with drug resistance and such indels should be identified and reported for both HIVDR surveillance and clinical monitoring purposes 22, 23, 24, 25. Identification of such indels at the final HIVDR reporting stage is a relevant outcome of this alignment and reference mapping step. Indel management strategies differ among existing pipelines (Table 1). While several pipelines claim to accommodate indels in variant calling and DRM detection, pipelines that use NGS short‐read aligners such as bowtie2 26 may not adequately address such needs, since short‐read aligners cannot straightforwardly be used to capture the effect of indels on the resulting coding sequence. Other approaches that perform haplotype phasing or that incorporate codon‐aware alignment strategies may be needed to reliably detect known HIVDR‐associated indels, but further evaluation is needed.
3. “**HIV variant calling and variant QC**” imposes additional stringency on the calling of variants, which is especially important when minority variants are concerned. NGS errors may arise at multiple points during sample processing (e.g. nucleic acid extraction, reverse transcription, PCR, template amplicon preparation for NGS and NGS sequencing) and NGS data processing 27. The gross error rates generated from short‐read NGS platforms ranges from approximately 1 to 10 errors per 1000 bases leading to increased false positive detection of minority variants when their prevalence falls below approximately 1% 13, 28, 29, 30. The additional variant QC strategies significantly improve the reliability of calling variants of low abundance, undetectable by Sanger sequencing. It is acknowledged that the threshold of minority variant frequency considered to be clinically relevant remains debatable 31.
4. “**NGS HIVDR interpretation and reporting**” is the only component designed specifically for HIVDR application, while all other blocks and associated strategies may find broader application, especially for genomic sequence analysis of microbes harbouring high genomic diversity, similar to HIV. This specific element of the pipeline streamlines the strategies to convert valid NGS‐derived amino acid variant data into end‐user‐interpretable HIVDR results. Two HIVDR report formats are recommended in this Consensus for addressing needs of either research‐oriented projects (a *comprehensive report*) or clinically oriented testing (a *concise report*). Ultimately, a customizable HIVDR reporting strategy is preferred for an optimal pipeline, allowing the users to construct a report of their preference. To facilitate comparisons and merging of data from different pipelines, a new standard amino acid variant file (aavf) format has been proposed (Appendix 1, https://github.com/winhiv/aavf-spec). Based on the variant call format (vcf) standard that has been universally adopted for recording nucleotide variants, the aavf report provides a compact summary of the amino acid variation obtained by conceptual translation of the NGS read pileup across the examined region of the HIV genome. It also contains information on the frequencies of matching codons (wild type or mutant), quality of the variant calling as well as the coverage of relevant loci. Although the specification is designed to fully accommodate the requirements for reporting of NGS‐based HIVDR testing, it is still suitably generic to serve as a general purpose file format for reporting amino acid variants for broader applications. A tool suite to parse aavf format is available at https://github.com/winhiv/PyAAVF.
5. “**General analysis data management**” deals with issues that concern both the data generator and the analysis provider, to protect the best interests of both parties, including formats and contents for data storage, software versioning, information traceability and data ownership policies.

This symposium was held at a time when NGS for HIVDR genotyping is increasingly being adopted by many laboratories for research, surveillance and clinical monitoring purposes. Although the functionalities and assembly of bioinformatics strategies applied in different pipelines vary, they share a common objective. The Winnipeg Consensus addresses the urgent needs for and starts the process of standardization of NGS HIVDR data analysis pipelines. It is noteworthy that most of the bioinformatics strategies described in the Winnipeg Consensus have already been incorporated in three of the assessed pipelines, which explains the high concordance among these pipelines when the same data sets were analysed 32. Although minor differences currently exist among PASeq, HyDRA and MiCall regarding the data processing procedures and reporting strategies, preliminary data suggests that these pipelines are largely interchangeable especially when only HIVDR mutations present at ≥5% are of interest 32.

An additional important outcome of this symposium was a consensus that a well‐characterized NGS HIVDR “dry panel” should be constructed in support of both pipeline development and validation applications. Such a dry panel would consist of a variety of simulated data files as well as empirical data sets derived from plasmids, artificial plasmid mixtures and patient specimens. It should also cover all major HIV‐1 subtypes and signature DRMs at a wide range of frequencies, allowing the flexibility for end‐users to customize panels based on their needs. Such a comprehensive panel is currently under construction by the symposium participant teams and will become freely accessible to the public once established. In fact, a subset of the dry panel has already been used for a comparison of PASeq, HyDRA and MiCall 32.

Additional NGS HIVDR assay comparative assessment strategies, such as parallel testing of the same plasma specimens in different laboratories followed by analysis of the raw NGS data from each laboratory using all available pipelines, are also underway. This is in collaboration with the Virology Quality Assurance (VQA) programme supported by the Division of AIDS at the National Institutes of Health, USA, which provides quality assurance support for HIVDR laboratories worldwide 33.

It is acknowledged that some limitations exist in the Winnipeg Consensus, including: (1) it only addresses strategic issues concerning NGS data processing and subsequent report accuracy. Errors arising from pre‐analytical procedures remain to be minimized through comprehensive protocol validations 34; (2) strategies described here ensure the quality of minority variant detection and reporting based solely on the input NGS data, thus assuming that the applied NGS reads directly represent the intrahost viral quasispecies. Understandably, the sensitivity and accuracy of NGS in minority variant quantification are inherently dependent on the initial HIV RNA template input, which in turn is defined by specimen characteristics and assay designs such as viral load, specimen volume processed, fraction of extracted nucleic acids used for RT‐PCR, efficiency of RNA to DNA conversion and evenness of PCR amplification for HIV templates present in the specimen. Related accuracy limitations might be partially addressed using more sophisticated experimental designs such as primerID which is likely beneficial for research purposes, but not yet proven to be necessary for routine clinical use and hence not dealt with in this consensus 13, 35, 36, 37, 38; and (3) it was developed primarily based on processing of data from Illumina technology, which is currently the most widely used, but not the only platform for NGS HIVDR^39^. Therefore, while Winnipeg Consensus principles apply to other NGS platforms, their exact implementation into data analysis pipelines will need to consider the platform‐specific characteristics and sequence error profiles for optimal results 20.

## 3 Conclusions

In conclusion, we present here the Winnipeg Consensus on bioinformatic strategies for NGS HIVDR data processing. This consensus may serve as an initial baseline to standardize NGS data analysis with a specific focus on HIVDR genotyping, and inform the refinement of existing pipelines and those still in development. This initiative and its subsequent activities may help make such technologies routine for both research and clinical HIVDR monitoring purposes, and may serve as a useful starting point for further developing of NGS analysis pipelines with similar and alternative intended applications.

## Competing interests

The authors have no competing interests to declare.

## Authors’ contributions

HJ, RP, PS, RH, GVD, RK and MNJ conceived and initiated the project. HJ, MNJ, NP, CJB and RK drafted this manuscript. All authors participated in the Winnipeg symposium and contributed to the manuscript revisions. MNJ, EE, CJB, MH, ERL, RC and EM led the discussions on varied bioinformatics issues at the symposium and summarized the consensus on the corresponding topics that were presented here. All authors contributed significantly to this study and have reviewed and approved the final version.
